# Refractory Multivessel Coronary Artery Spasm Unresponsive to Renal Sympathetic Denervation, with Recurrent Transient ST-Segment Elevation and an Episode of Accelerated Idioventricular Rhythm: A Case Report

**DOI:** 10.3390/reports9040338

**Published:** 2026-10-07

**Authors:** Al-Naser Al Bahlani, Hiba Saadeddin, Ayman Al Husini, Mubarak Al Dosari, Hussein Hado

**Affiliations:** Heart Health Center, Adult Cardiology Department, King Saud Medical City, Riyadh 12742, Saudi Arabia; hibakhalifa82@hotmail.com (H.S.);

**Keywords:** coronary artery spasm, vasospastic angina, multivessel coronary spasm, transient ST-segment elevation, accelerated idioventricular rhythm, refractory vasospastic angina, renal sympathetic denervation, case report

## Abstract

**Background and Clinical Significance:** Multivessel coronary artery spasm (CAS) is an uncommon, high-risk form of vasospastic angina that can produce transient ST-segment elevation mimicking acute myocardial infarction. Evidence to guide management when calcium channel blockers and nitrates fail is scarce, and renal sympathetic denervation (RSD) has been reported in very few patients; **Case Presentation:** A 53-year-old man with hypertension, type 2 diabetes, and dyslipidemia presented with nocturnal chest pain and inferior ST-segment elevation. Coronary angiography showed severe spontaneous spasm of the right coronary and left anterior descending arteries that resolved completely with intracoronary nitrate; the coronary arteries were otherwise angiographically normal. High-sensitivity cardiac troponin I remained below 10 ng/L (reference 0–34 ng/L), and echocardiography was normal, fulfilling the criteria for definite vasospastic angina without myocardial infarction. The same two-vessel spasm was documented at a second angiogram three months later. Despite maximally tolerated dual calcium channel blockade, nitrates, and statin therapy, he required more than ten further admissions. A self-terminating accelerated idioventricular rhythm lasting about two minutes was recorded. No syncope or malignant arrhythmia occurred, and an electrophysiology review found no current indication for an implantable cardioverter–defibrillator. Radiofrequency RSD was performed off-label for refractory symptoms without complications, but at six months, his symptoms and admissions were unchanged; **Conclusions:** Our case highlights the diagnostic and therapeutic difficulties in refractory CAS. Accelerated idioventricular rhythm after relief of spasm is a reperfusion rhythm and does not by itself indicate device therapy. In this patient, RSD did not control refractory multivessel CAS, a negative observation that adds to the very limited evidence on autonomic modulation in this setting.

## 1. Introduction and Clinical Significance

Coronary artery spasm (CAS) is a transient, intense constriction of an epicardial coronary artery that causes myocardial ischemia. Its manifestations range from rest angina to acute coronary syndromes, ventricular arrhythmias, syncope, and sudden cardiac death [1,2]. When spasm is prolonged, it can cause myocardial infarction in the absence of obstructive coronary disease (myocardial infarction with non-obstructive coronary arteries, MINOCA) [3,4]. More often, the ischemia is brief, and no infarction occurs; this presentation is vasospastic angina, which belongs to the spectrum of ischemia with non-obstructive coronary arteries (INOCA) [5,6,7].

Most patients with CAS respond to calcium channel blockers and nitrates, but a subset continues to have episodes despite combined vasodilator therapy and is considered to have refractory disease [8]. Multivessel spasm identifies a higher-risk group: it is one of the predictors in the Japanese Coronary Spasm Association (JCSA) risk score for major adverse cardiac events [9].

The pathophysiology of CAS is multifactorial. Endothelial dysfunction, hyperreactivity of vascular smooth muscle, inflammation, and autonomic imbalance lower the threshold for coronary constriction, and activation of the Rho-kinase pathway has been implicated in sustained spasm [3,8,10].

Accelerated idioventricular rhythm (AIVR) is classically a reperfusion rhythm, seen after restoration of coronary flow in acute myocardial infarction [11,12]. Relief of coronary spasm restores flow in the same abrupt way, yet AIVR in this setting has rarely been reported.

Treatment options beyond calcium channel blockers and nitrates include nicorandil, statins, and Rho-kinase inhibition, each supported by limited evidence [2,8,10]. Autonomic modulation by renal sympathetic denervation (RSD) has been described in one case report and one ten-patient pilot study from a single center [13,14].

We report a patient with definite vasospastic angina due to spontaneous spasm of the right coronary artery (RCA) and left anterior descending artery (LAD), recurrent transient ST-segment elevation without myocardial infarction, AIVR after relief of spasm, and persistent episodes despite maximally tolerated medical therapy and RSD.

## 2. Case Presentation

### 2.1. Index Presentation

A 53-year-old man with hypertension, type 2 diabetes mellitus, and dyslipidemia presented to the emergency department with sudden, severe left-sided chest pain that woke him from sleep. The pain radiated to the left arm and was accompanied by diaphoresis. He had never smoked and did not use alcohol or recreational drugs. There was no family history of premature coronary artery disease or sudden cardiac death.

**Figure 1:**
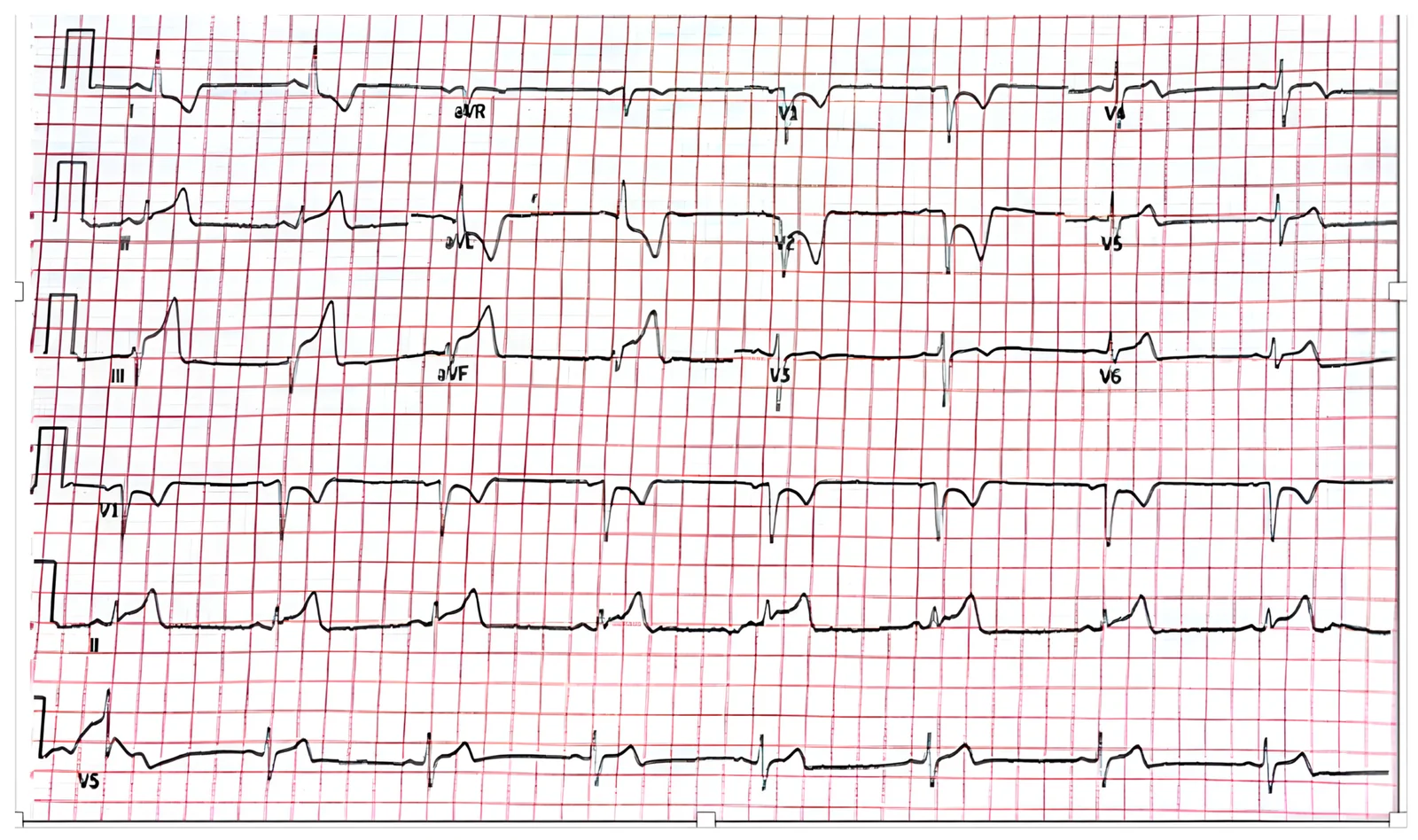
Twelve-lead electrocardiogram at the index presentation showing sinus bradycardia with ST-segment elevation in leads II, III, and aVF, and ST-segment depression in leads I, aVL, and V1–V3.

On arrival, he was in severe pain but hemodynamically stable, and cardiovascular and respiratory examinations were unremarkable. The electrocardiogram (ECG) showed sinus bradycardia with ST-segment elevation in the inferior leads (Figure 1); posterior leads confirmed posterior involvement. The working diagnosis was acute inferior ST-segment elevation myocardial infarction.

**Figure 2:**
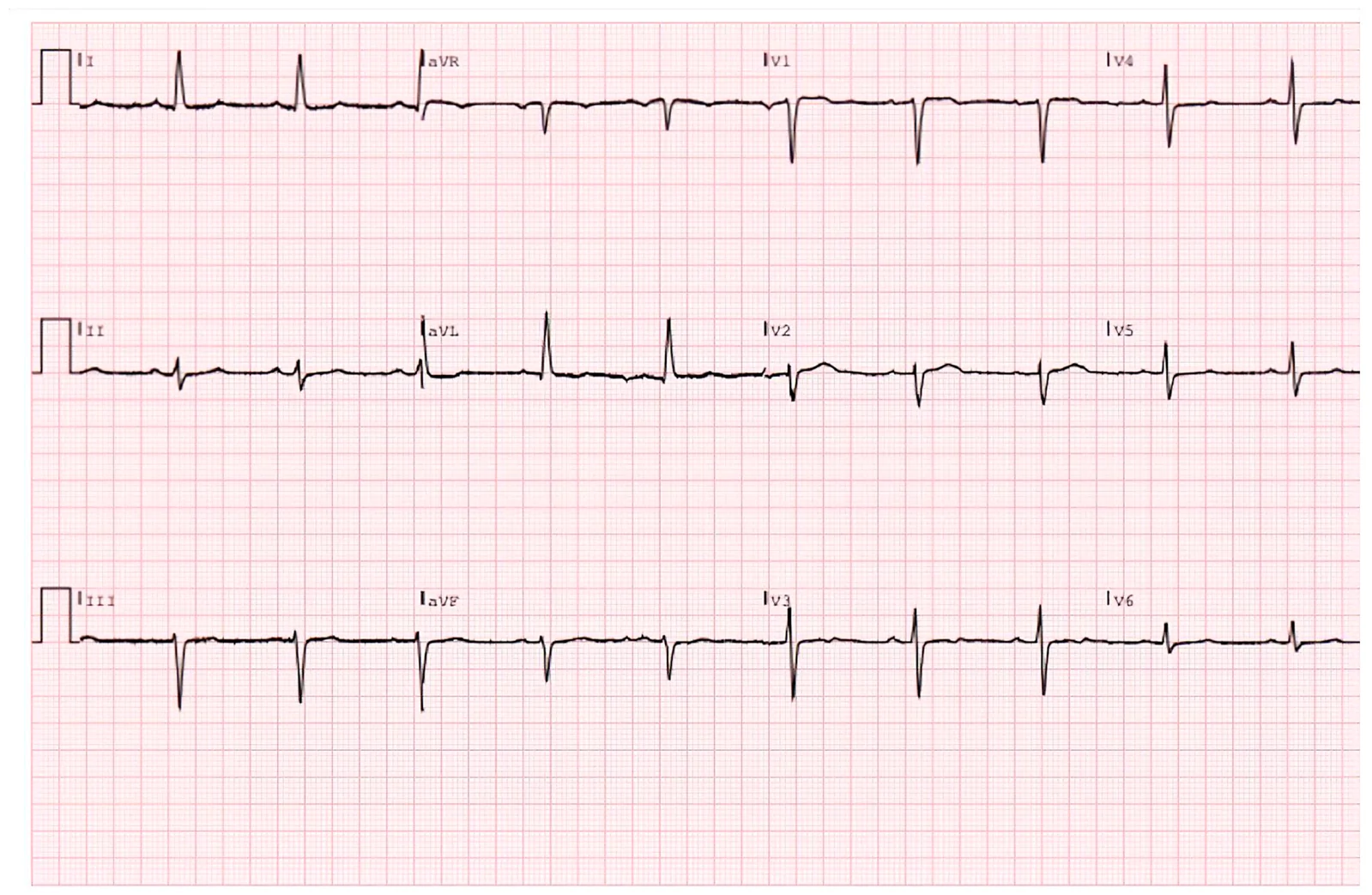
Twelve-lead electrocardiogram recorded after resolution of the index episode, showing sinus rhythm without ST-segment elevation.

**Figure 3:**
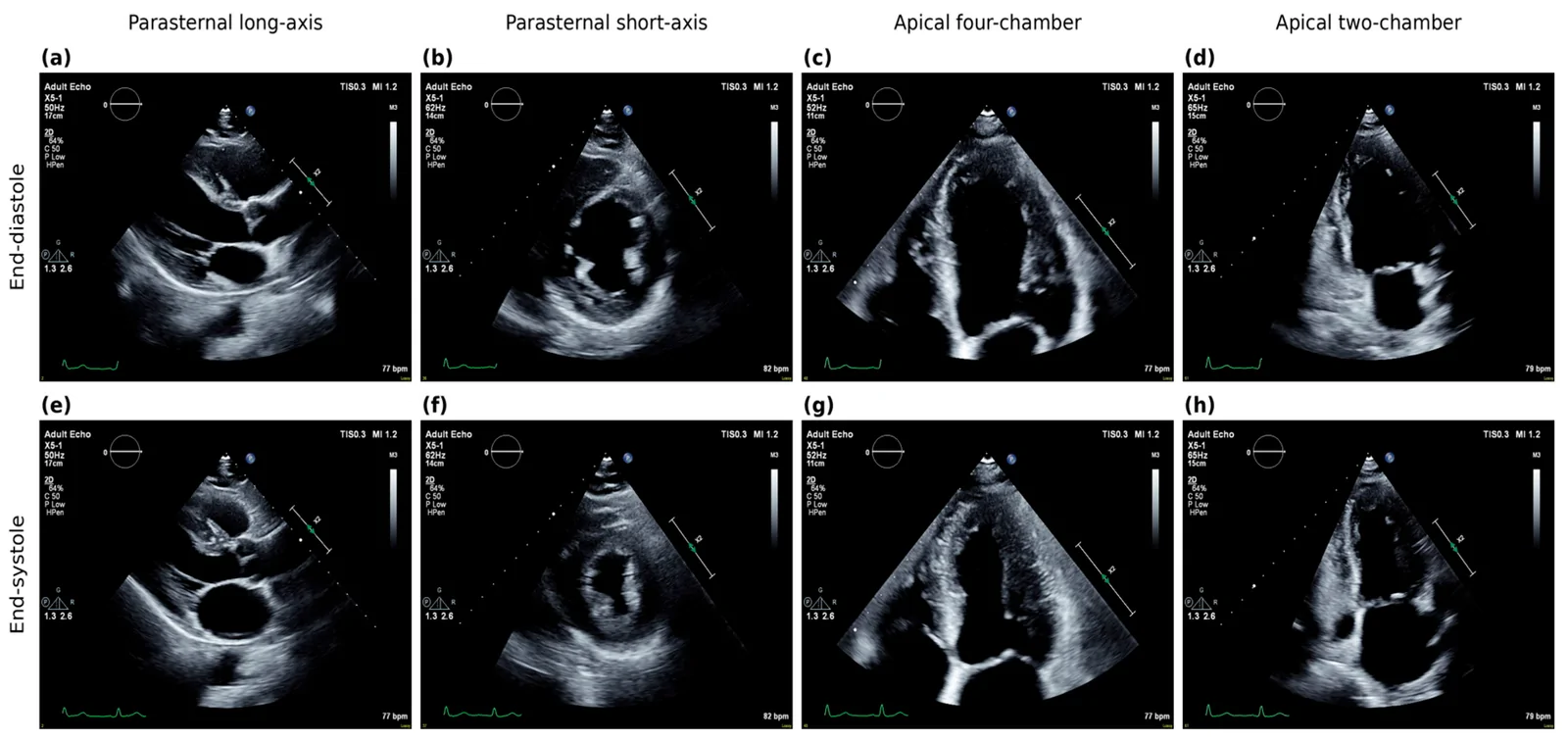
Transthoracic echocardiography at end-diastole (**a**–**d**) and end-systole (**e**–**h**) in the parasternal long-axis (**a**,**e**), parasternal short-axis at the mid-papillary level (**b**,**f**), apical four-chamber (**c**,**g**), and apical two-chamber (**d**,**h**) views, showing normal left ventricular wall thickness and uniform systolic wall thickening without regional wall motion abnormality.

Emergency coronary angiography showed severe spontaneous spasm (>90% luminal narrowing) of both the RCA and the LAD, with otherwise angiographically normal coronary arteries. Intracoronary nitrate produced complete resolution of the narrowing and restored normal vessel caliber (Videos S1–S4). The chest pain resolved, and a repeat ECG showed resolution of the ST-segment elevation with sinus rhythm (Figure 2). High-sensitivity cardiac troponin I (hs-cTnI) remained below 10 ng/L on serial testing (reference range 0–34 ng/L). Transthoracic echocardiography showed normal biventricular systolic function with no regional wall motion abnormality (Figure 3). None of the criteria for myocardial infarction were therefore met [4], and the diagnosis was vasospastic angina due to multivessel CAS.

The patient met all three Coronary Vasomotor Disorders International Study Group (COVADIS) criteria for vasospastic angina (Table 1) [5].

### 2.2. Recurrence and Escalation of Medical Therapy

**Figure 4:**
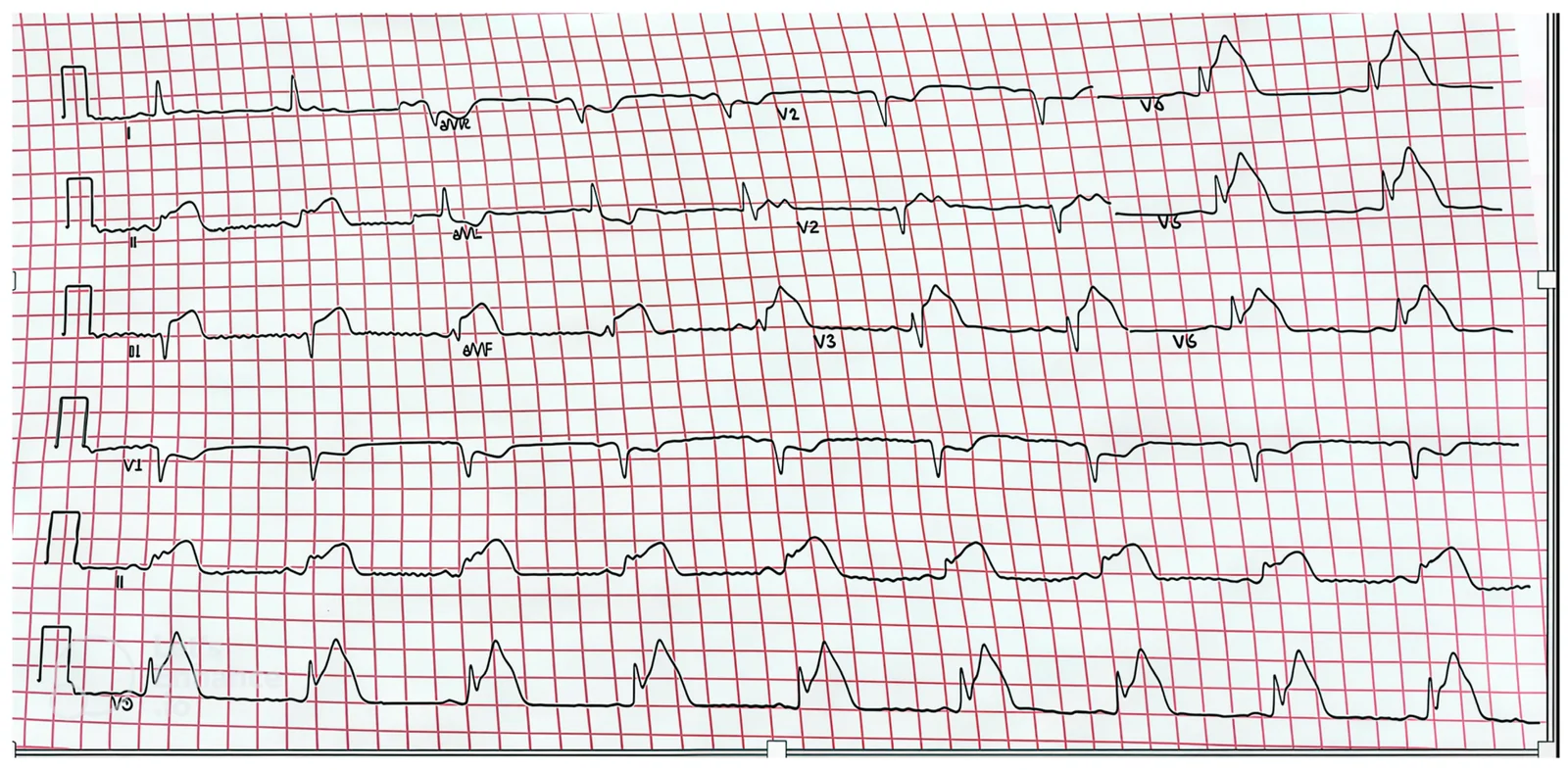
Twelve-lead electrocardiogram at the second presentation showing ST-segment elevation in leads II, III, aVF, and V3–V6.

**Figure 5:**
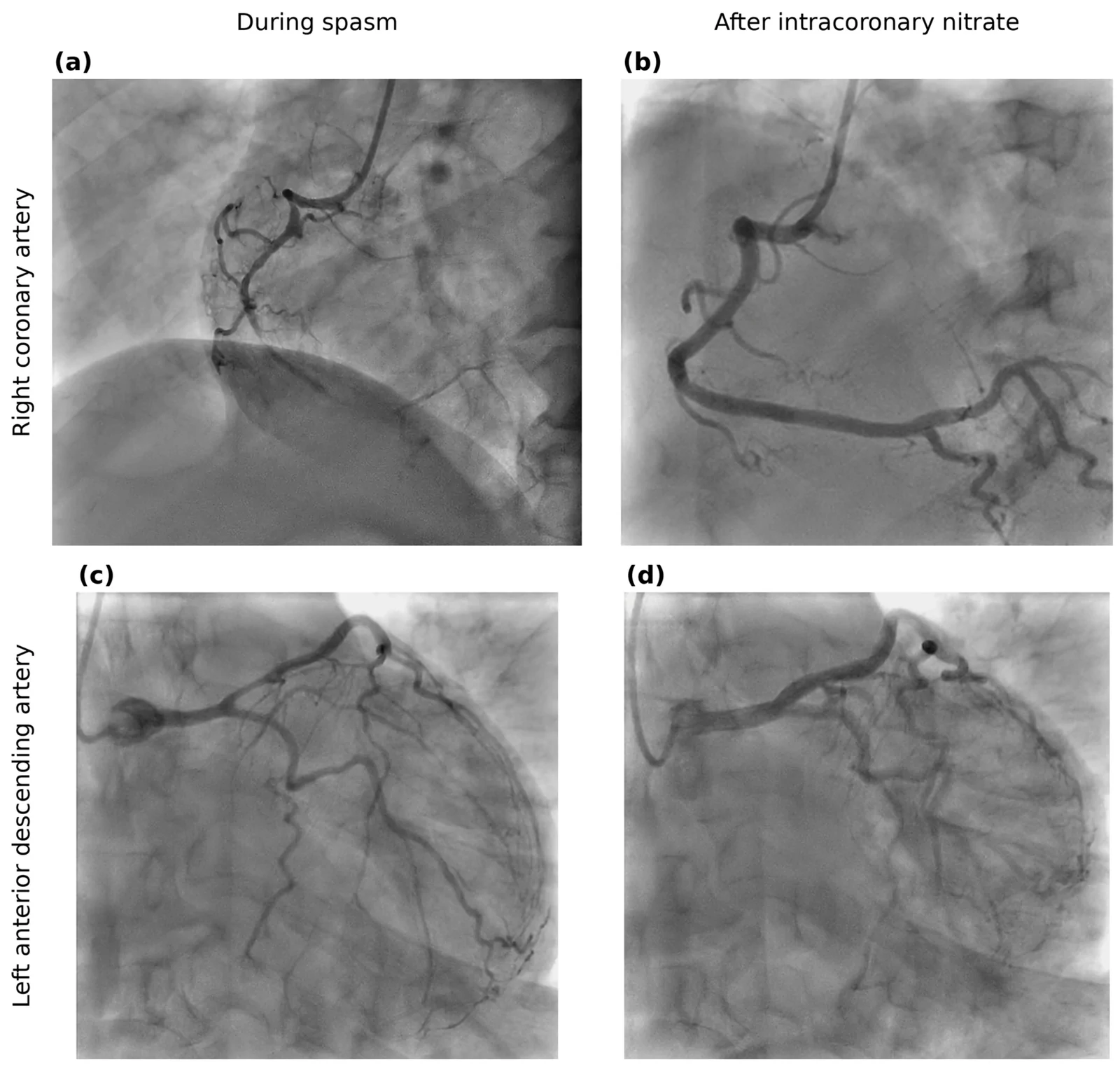
Coronary angiography during spontaneous spasm and after intracoronary nitrate. (**a**) Right coronary artery during spasm, showing severe narrowing at multiple sites. (**b**) Right coronary artery after intracoronary nitrate, with restoration of normal caliber. (**c**) Left anterior descending artery during spasm. (**d**) Left anterior descending artery after intracoronary nitrate.

Three months later, while taking his prescribed therapy, he presented again with chest pain identical to the index episode. Blood pressure was 80/40 mmHg. The ECG showed ST-segment elevation in the inferior and anterolateral leads (Figure 4), and hs-cTnI again remained below 10 ng/L. Repeat coronary angiography showed the same pattern of severe spontaneous spasm of the RCA and LAD, with complete resolution after intracoronary nitrate (Figure 5). Echocardiography was again normal. The hypotension responded to intravenous fluids and was attributed to vasodilator therapy and not to ischemic ventricular dysfunction.

Medical therapy was escalated to a maximally tolerated combination of calcium channel blockade with a dihydropyridine and a non-dihydropyridine agent, together with a long-acting nitrate and short-acting nitrate as required. He was also taking a statin for dyslipidemia and diabetes. Further dose escalation was limited by hypotension. Nicorandil is not available at our center.

Despite reported adherence, vasospastic episodes continued and led to more than ten further hospital admissions. The clinical course is summarized in Table 2.

### 2.3. Accelerated Idioventricular Rhythm and Arrhythmic Risk Assessment

**Figure 6:**
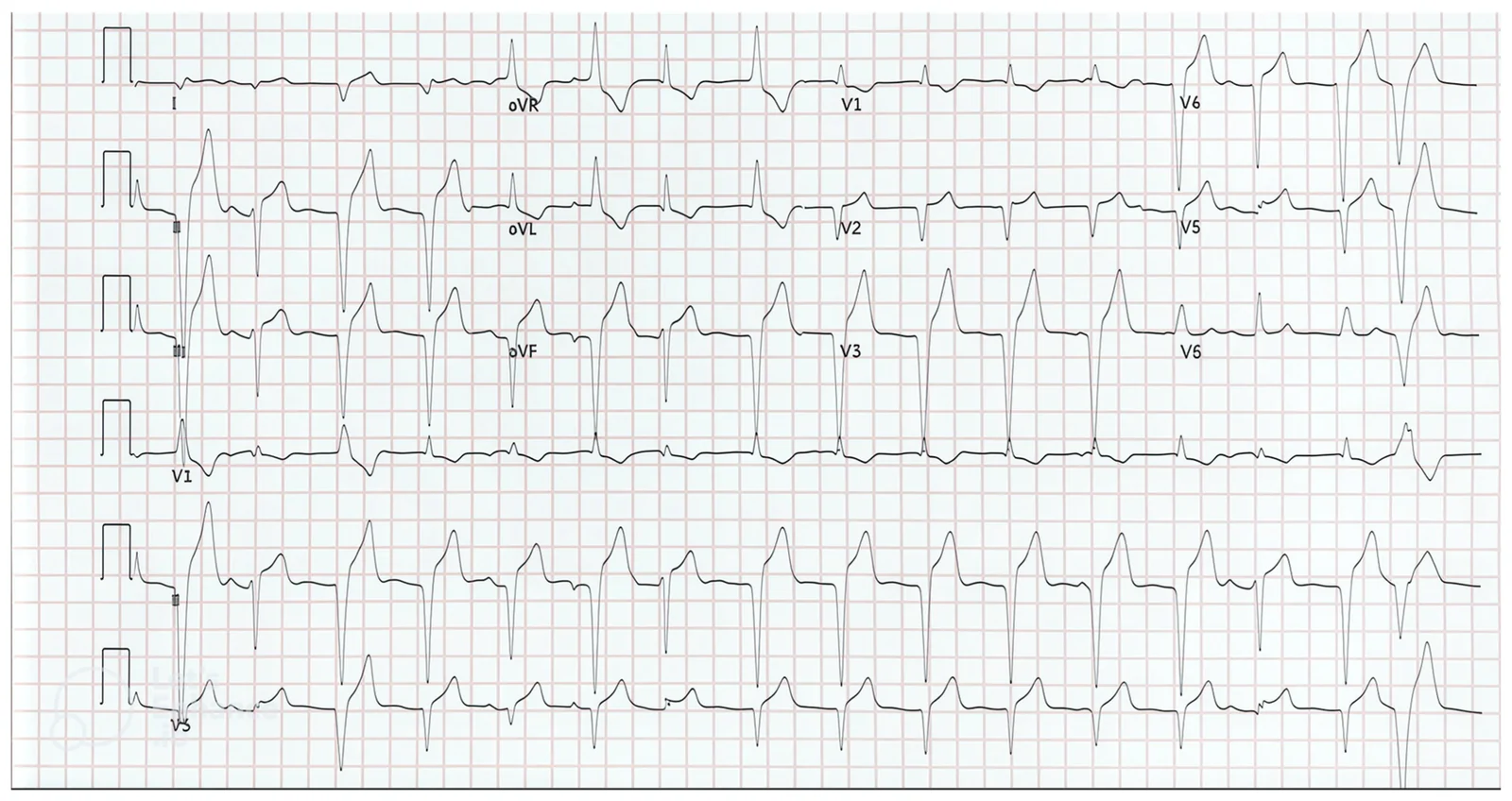
Twelve-lead electrocardiogram showing accelerated idioventricular rhythm at a ventricular rate of 100 beats per minute, recorded shortly after spontaneous relief of a vasospastic episode.

During one admission for severe chest pain with transient ischemic ECG changes, the pain and ECG changes resolved spontaneously. Shortly afterwards, telemetry showed a wide-complex rhythm, which a 12-lead ECG confirmed as AIVR (Figure 6). It lasted about two minutes and terminated spontaneously. He remained hemodynamically stable and needed no treatment. Serum potassium and magnesium were within normal limits. The rhythm was interpreted as a reperfusion rhythm following relief of spasm.

Because of the multivessel spasm and recurrent events, the electrophysiology team reviewed him. He had no history of syncope or presyncope, no cardiac arrest, and no sustained or non-sustained ventricular tachycardia or ventricular fibrillation on telemetry during his admissions; left ventricular ejection fraction was normal. The team concluded that an implantable cardioverter–defibrillator (ICD) was not currently indicated and that the decision should be revisited if syncope or a malignant ventricular arrhythmia occurs.

### 2.4. Renal Sympathetic Denervation and Follow-Up

After about one year of recurrent episodes on maximally tolerated therapy, catheter-based radiofrequency RSD was offered as a treatment strategy after multidisciplinary discussion. The indication was control of refractory spasm and symptoms; he did not have resistant hypertension. He was counseled that this was an off-label use supported by very limited evidence, and he consented. The procedure was completed without immediate complications.

At six months after RSD, his symptoms were unchanged: he continued to have vasospastic episodes requiring hospital admission, with no clear benefit from the procedure. He remains under regular follow-up, still has recurrent chest pain that responds partially to short-acting nitrates, and has had no further arrhythmia recorded.

## 3. Discussion

### 3.1. Diagnostic Classification: Vasospastic Angina, Not Myocardial Infarction

Each of this patient’s presentations looked like an ST-segment elevation myocardial infarction at first contact, but none was an infarction. The Fourth Universal Definition requires a rise and/or fall of cardiac troponin with at least one value above the 99th percentile upper reference limit [4]. His hs-cTnI stayed below that limit on serial testing, the ST-segment elevation resolved with nitrates, and there was no wall motion abnormality. The terms ST-segment elevation myocardial infarction and MINOCA therefore do not apply. The correct description is vasospastic angina with transient ST-segment elevation, within the spectrum of INOCA [5,6]. He fulfilled all three COVADIS criteria (Table 1), which establishes definite vasospastic angina without the need for provocation testing [5].

The distinction has practical consequences. A label of myocardial infarction leads to treatments designed for atherothrombosis, including beta-blockers, which can aggravate spasm and are themselves a predictor of adverse events in the JCSA score [8,9]. The normal troponin despite marked ST-segment elevation and two-vessel spasm also shows that severe electrical changes can accompany ischemia that is too brief to cause necrosis.

### 3.2. Multivessel Spasm and Refractoriness

Multivessel spasm marks more severe disease. The JCSA score comprises seven predictors: history of out-of-hospital cardiac arrest, smoking, angina at rest alone, organic coronary stenosis, multivessel spasm, ST-segment elevation during angina, and beta-blocker use [9]. Our patient had three of them: multivessel spasm, ST-segment elevation during attacks, and angina at rest. The same two arteries were involved on two angiograms three months apart, which points to a persistent vasomotor abnormality and not an isolated episode. He had hypertension, diabetes, and dyslipidemia but had never smoked, the risk factor most consistently linked to CAS [1].

Why some patients do not respond to vasodilators has not been established. Rho-kinase activation increases the calcium sensitivity of vascular smooth muscle and may sustain constriction that calcium channel blockade does not fully reverse [8]. Endothelial dysfunction, inflammation, and autonomic influences probably act together [3,10]. In this patient, recurrence on dual calcium channel blockade, nitrates, and a statin, and the lack of response to RSD, are consistent with a predominantly intrinsic vascular abnormality, although a single case cannot establish the mechanism.

### 3.3. Accelerated Idioventricular Rhythm After Relief of Spasm

Accelerated idioventricular rhythm is an ectopic ventricular rhythm faster than the intrinsic ventricular escape rate but slower than ventricular tachycardia, with gradual onset and termination [11]. It is usually well tolerated and requires no treatment [11,12]. It is attributed to enhanced automaticity of Purkinje fibers or ventricular myocardium when flow is restored to ischemic tissue, and it becomes manifest when its rate exceeds the sinus rate [11]. Relief of spasm is a form of reperfusion. Two factors probably favored the rhythm in our patient. First, coronary flow returned abruptly when the spasm resolved spontaneously. Second, his sinus rate was slow because he had sinus bradycardia at presentation and was taking a non-dihydropyridine calcium channel blocker, and a slow sinus rate allows a ventricular focus to take over. Normal potassium and magnesium made an electrolyte cause unlikely.

Ventricular arrhythmias in variant angina occur in both phases of an attack. In 56 patients studied by Previtali et al., about half had ventricular tachyarrhythmias; these arose during the occlusive phase in 23 patients and during reperfusion in 10, and reperfusion arrhythmias were associated with longer episodes and greater ST-segment elevation [15]. Reports of AIVR specifically are few (Table 3). Assefa et al. described a man with anterolateral ST-segment elevation and negative biomarkers in whom a slow ventricular rhythm followed an episode and ended after sublingual nitroglycerin [16]. Kim et al. reported AIVR that began one hour after a nitroglycerin infusion for RCA spasm with infarction and persisted for five days [17]. In both reports, the rhythm followed nitrate administration. In our patient, it followed spontaneous resolution of spasm, which indicates that the rhythm reflects reperfusion itself and not an effect of nitrates.

### 3.4. Arrhythmic Risk and Implantable Cardioverter–Defibrillator Candidacy

Accelerated idioventricular rhythm itself is benign and should not be counted as a marker of arrhythmic risk. The relevant question in a patient with refractory multivessel spasm is the risk of malignant ventricular arrhythmia. The 2022 European Society of Cardiology guidelines state that ICD implantation should be considered in survivors of sudden cardiac arrest with coronary artery spasm (class IIa) [18]. Our patient had no cardiac arrest, syncope, or ventricular tachyarrhythmia, and his ventricular function was normal, so he had no guideline indication for an ICD. This was the conclusion of the electrophysiology review. We nevertheless regard him as at higher risk than most patients with vasospastic angina because spasm is multivessel and incompletely controlled, both of which are associated with adverse events [9]. The hypotension at his second presentation responded to fluids and reflected vasodilator therapy; we do not consider it a marker of hemodynamically significant ischemia. The ICD decision will be revisited if syncope or a malignant arrhythmia occurs.

### 3.5. Renal Sympathetic Denervation: Rationale and Patient Selection

Renal sympathetic denervation is an established treatment option for hypertension; a European consensus statement notes that indications beyond hypertension remain to be investigated [19]. Its use in vasospastic angina rests on the role of autonomic imbalance in coronary vasomotion [3,10] and on two reports from one center. Feyz et al. described a normotensive man with ventricular fibrillation due to vasospastic angina who was free of angina and ventricular arrhythmia 12 months after RSD [13]. The same group then treated ten consecutive patients with refractory vasospastic angina; the Canadian Cardiovascular Society angina class improved from 3.0 to 1.8 at six months, and quality-of-life scores improved [14]. That study had important limitations, which its authors acknowledged: there was no sham control, so a placebo effect could not be excluded; anti-anginal drugs were changed in seven of the ten patients during follow-up; and cardiac sympathetic activity measured by iodine-123 meta-iodobenzylguanidine imaging did not change [14].

No validated criteria exist for selecting patients with vasospastic angina for RSD; the pilot study enrolled on the basis of refractory angina alone [14]. Our team required all of the following: (i) definite vasospastic angina by COVADIS criteria, with spontaneous multivessel spasm documented on two angiograms; (ii) no obstructive coronary artery disease; (iii) failure of maximally tolerated dual calcium channel blockade and nitrates, with a statin, and dose escalation limited by hypotension; (iv) a high event burden, with more than ten admissions; (v) unavailability of nicorandil; and (vi) multidisciplinary agreement and informed consent after counseling on the off-label nature of the procedure. Blood pressure was not an indication.

Six months after RSD, his symptoms and admissions remained unchanged. This non-response is consistent with the pilot study’s own finding that cardiac sympathetic activity was unchanged after RSD, and it raises the possibility that the symptomatic benefit reported there was partly a placebo effect. Other explanations are possible: denervation may have been incomplete, since no procedural marker of success exists; we used radiofrequency energy, whereas the published reports used an ultrasound system [13,14]; and the dominant mechanism in this patient may not be sympathetic. None of the ten pilot patients had diabetes, whereas our patient did [14]; whether this matters is unknown. Our observation does not show that RSD is ineffective in vasospastic angina, but it argues against offering it outside a controlled study.

### 3.6. Other Treatment Options

*Statins:* In a randomized trial of 64 patients, adding fluvastatin to a calcium channel blocker reduced acetylcholine-inducible spasm at six months [20]. Our patient was already taking a statin, so this option had been used. *Nicorandil:* This potassium-channel opener with nitrate-like action is used as add-on therapy for refractory spasm, largely on the basis of Japanese experience [2,8]; it is not available at our center, which is a real constraint on what refractory means in this report. Rho-kinase inhibition with fasudil has been used for refractory spasm, but the evidence is limited to small studies [8].

*Stellate ganglion block and surgical sympathectomy:* Evidence is limited to case reports and small series. Yoshida et al. performed bilateral endoscopic thoracic sympathectomy in five men with refractory vasospastic angina [21]; Cardona-Guarache et al. reported thoracic sympathectomy for severe refractory multivessel spasm [22]; Wu et al. used a stellate ganglion block as a therapeutic trial before bilateral thoracoscopic sympathectomy, with no recurrence of spasm [23]; and Malik et al. reported that sympathetic denervation abolished recurrent ventricular fibrillation and intractable coronary spasm [24]. Like RSD, these are autonomic interventions without controlled data. We chose RSD first because it is percutaneous and the expertise was available locally, whereas sympathectomy requires thoracoscopic surgery. After the non-response to RSD, we have not pursued a second autonomic intervention, although a temporary stellate ganglion block remains a possible therapeutic trial if his symptoms worsen.

*Percutaneous coronary intervention:* Stenting has been reported for focal spasm that does not respond to drugs, but spasm can recur in other segments, and medical therapy must be continued [2,8]. Our patient had diffuse spasm of two vessels with otherwise normal arteries and no focal target, so stenting was not considered.

### 3.7. Limitations

This is a single case, and no causal inference can be drawn about the effect of RSD. Follow-up after RSD is limited to six months. The frequency of episodes before and after RSD was not recorded prospectively, and no measure of sympathetic activity or of the completeness of denervation was obtained. Spasm severity was estimated visually and not by quantitative coronary angiography. Intracoronary imaging and invasive coronary function testing were not performed, so coexisting microvascular dysfunction or angiographically silent atherosclerosis cannot be excluded. Refractoriness was defined against the drugs available to us; nicorandil and other second-line agents were not tried. Rhythm monitoring was limited to in-hospital telemetry, so arrhythmias outside the hospital may have been missed. Genetic testing was not performed.

## 4. Conclusions

Three lessons follow from this case. Transient, nitrate-reversible ST-segment elevation with normal high-sensitivity troponin is vasospastic angina and should be named as such, with the COVADIS criteria used to make the diagnosis explicit. Accelerated idioventricular rhythm after relief of spasm is a reperfusion rhythm; arrhythmic risk and ICD candidacy should be judged on syncope, malignant arrhythmia, and cardiac arrest, not on AIVR. Finally, RSD did not control refractory multivessel spasm in this patient, and autonomic interventions for vasospastic angina should be evaluated in controlled studies before they are offered in routine practice.

## Figures and Tables

**Table 1:** Diagnostic criteria for vasospastic angina of the Coronary Vasomotor Disorders International Study Group (COVADIS) [5] and the corresponding findings in this patient.

Criterion	Finding in This Patient
1. Nitrate-responsive angina with at least one of the following: rest angina (especially at night or early morning), marked diurnal variation in exercise tolerance, precipitation by hyperventilation, or suppression by calcium channel blockers	Met. Recurrent angina at rest, with onset during sleep at the index episode, relieved by nitrates
2. Transient ischemic ECG changes during a spontaneous episode: ST-segment elevation or depression ≥ 0.1 mV, or new negative U waves, in at least two contiguous leads	Met. Transient ST-segment elevation in the inferior leads (Figure 1) and in the inferior and anterolateral leads (Figure 4), with resolution after nitrates
3. Coronary artery spasm: transient total or subtotal occlusion (>90% constriction) with angina and ischemic ECG changes, occurring spontaneously or on provocation	Met. Spontaneous spasm with >90% narrowing of the RCA and LAD on two angiograms three months apart, reversed by intracoronary nitrate
Classification	Definite vasospastic angina

COVADIS, Coronary Vasomotor Disorders International Study Group; LAD, left anterior descending artery; RCA, right coronary artery. Definite vasospastic angina requires nitrate-responsive angina together with either the electrocardiographic or the angiographic criterion.

**Table 2:** Timeline of the clinical course.

Time Point	Event
Index admission	Nocturnal chest pain with inferior ST-segment elevation; spontaneous spasm (>90%) of the RCA and LAD reversed by intracoronary nitrate; hs-cTnI < 10 ng/L; normal echocardiogram; calcium channel blocker and nitrate therapy started
3 months	Recurrent pain with inferior and anterolateral ST-segment elevation; fluid-responsive hypotension (80/40 mmHg); same two-vessel spasm on repeat angiography; hs-cTnI < 10 ng/L; therapy escalated to dual calcium channel blockade plus nitrates
First year	More than ten further admissions for vasospastic episodes despite maximally tolerated therapy; one episode followed by AIVR lasting about two minutes; electrophysiology review: no current ICD indication
About 1 year	Radiofrequency RSD performed off-label for refractory symptoms; no immediate complications
6 months after RSD	Symptoms unchanged, with no clear benefit; recurrent admissions continue; chest pain partially responsive to short-acting nitrates; no further arrhythmia recorded

AIVR, accelerated idioventricular rhythm;; hs-cTnI, high-sensitivity cardiac troponin I; ICD, implantable cardioverter–defibrillator; LAD, left anterior descending artery; RCA, right coronary artery; RSD, renal sympathetic denervation.

**Table 3:** Reported cases of accelerated idioventricular rhythm associated with coronary artery spasm.

	Assefa et al. [16]	Kim et al. [17]	Present Case
Patient	57-year-old man	56-year-old man	53-year-old man
Territory	Anterolateral ST-segment elevation; unremarkable angiogram	RCA spasm; inferior ST-segment elevation	RCA and LAD spasm; inferior and anterolateral ST-segment elevation
Myocardial infarction	No (biomarkers negative)	Yes (spasm-induced)	No (hs-cTnI < 10 ng/L)
Onset of AIVR	After an episode of ST-segment elevation	One hour after nitroglycerin infusion	Shortly after spontaneous resolution of an episode
Duration and outcome	Brief; ended after sublingual nitroglycerin	Five days; resolved spontaneously	About two minutes; resolved spontaneously

AIVR, accelerated idioventricular rhythm; LAD, left anterior descending artery; RCA, right coronary artery.

## Data Availability

The original contributions presented in this study are included in the article and Supplementary Materials. Further inquiries can be directed to the corresponding author.

## Supplementary Materials

The following supporting information can be downloaded at https://www.mdpi.com/article/10.3390/reports9040338/s1, Video S1: Right coronary artery during spontaneous spasm; Video S2: Right coronary artery after intracoronary nitrate; Video S3: Left anterior descending artery during spontaneous spasm; Video S4: Left anterior descending artery after intracoronary nitrate.
